# Identification through the Teeth

**Published:** 1905-02

**Authors:** 


### IDENTIFICATION THROUGH THE TEETH.

Upon another page will be found a communication from the chief of police of Colorado Springs, requesting dentists generally to assist him in the identification of the body of a woman found dead in that vicinity. This identification it is hoped may be secured through an examination of the accompanying diagram of the teeth. As this woman seems to have been in charge of a competent dentist, it ought not to be difficult for one who has kept careful records to identify the work.

The fact that when all other means of identification have been exhausted, the examination of the teeth has alone accomplished the solution of the problem is of great importance. The cases on record are many in proof of this, but the celebrated case of Webster-Parkman will be remembered by many, where Dr. Keep was enabled to identify the remnants of the incinerated jaw as that of Dr. Parkman, through the models he had kept, and it was principally on this evidence that Webster was convicted.

The possibility of being called to testify in similar cases should cause great care in keeping records in prosthetic work, as well as in operative procedures. This is certainly more carefully looked after at the present time than in former years, as the active practitioner has found it necessary for his own protection; hence the identification of the dead through the examination of the teeth has become part of dental jurisprudence, as officials recognize the importance of this class of evidence.

